# Generation of synthetic vascular organoids via orthogonal programming of human pluripotent stem cells

**DOI:** 10.1186/s13619-026-00298-6

**Published:** 2026-07-14

**Authors:** Yun Zhao, Mengze Sun, Kun Zhang, Juan M. Melero-Martin, Xi Wang, Kai Wang

**Affiliations:** 1 Department of Physiology and Pathophysiology, School of Basic Medical Sciences, State Key Laboratory of Vascular Homeostasis and Remodeling, Beijing Advanced Center of Cellular Homeostasis and Aging-Related Diseases, Clinical Stem Cell Research Center, Peking University Third Hospital, Peking University, Beijing, 100191 China; 2 Chinese Academy of Sciences Investment Management Co., Ltd., Beijing, 100080 China; 3 Department of Cardiac Surgery, Boston Children’s Hospital, Boston, MA 02115 USA; 4 Department of Surgery, Harvard Medical School, Boston, MA 02115 USA; 5 Harvard Stem Cell Institute, Cambridge, MA 02138 USA; 6 Department of Obstetrics and Gynecology, State Key Laboratory of Female Fertility Promotion, Peking University Third Hospital, Institute of Advanced Clinical Medicine, Peking University, Beijing, 100191 China

**Keywords:** Vascular organoid, Human pluripotent stem cell, ETV2, NKX3.1

## Abstract

The development of functional human vasculature is essential for tissue engineering, disease modeling, and regenerative medicine. Conventional differentiation protocols of vascular lineages often exhibit lineage heterogeneity and limited control over cellular ratios. Here, we describe a protocol for generating vascular organoids (VOs) via orthogonal forward programming of hPSCs. By utilizing doxycycline-inducible activation of the transcription factors ETV2 and NKX3.1, hPSCs are rapidly directed toward endothelial and mural cell lineages, respectively. This strategy enables the assembly of VOs with precisely tunable cellular compositions within six days. When combined with fluorescent reporter lines (*PECAM1-mRuby3* and *ACTA2-EGFP*), vascular networks can be visualized in real time without the need for tissue clearing or immunostaining. We detail procedures for stable cell line engineering, 3D organoid assembly, in vitro angiogenesis assays for drug screening, and in vivo transplantation under the mouse kidney capsule to form perfusable human vasculature. This platform provides a flexible, standardized, and scalable tool for investigating vascular biology, modeling inherited vasculopathies, and enhancing the vascularization of co-transplant tissues.

## Background

The vasculature is central to development, tissue repair, and disease (Carmeliet 2003; Augustin and Koh 2024), and human pluripotent stem cell (hPSC)-derived vascular models provide a valuable platform to study these processes in controlled experimental settings (Wimmer et al. 2019a, 2019b; Gong et al. 2025). Existing approaches to generate human vasculature from hPSCs include in vivo transplantation of hPSC-derived endothelial cells (ECs) with supporting stromal cells, as well as in vitro systems based on embryoid body (EB) differentiation and extracellular matrix (ECM) embedding (Wimmer et al. 2019a). While these methods have enabled the formation of functional and perfusable human vessels, they often involve prolonged differentiation, variable lineage specification efficiency and limited control over cellular composition, which can complicate experimental design and reproducibility. Another practical limitation of current vascular models is the visualization of vascular structures. Assessment of vascular network organization frequently relies on whole-mount immunostaining or tissue clearing, which are technically demanding and time-consuming, particularly for iterative optimization or screening-based assays.

Here, we present a doxycycline-inducible forward programming strategy for the rapid generation of vascular organoids (VOs) from hPSCs. Inducible activation of ETV2 and NKX3.1 enables robust and reproducible induction of ECs and mural cells (MCs), yielding organoids with tunable cellular composition within a short timeframe (Gong et al. 2025). By combining this system with fluorescent reporter hPSC lines, vascular networks can be directly visualized without additional clearing or staining steps (Zhao et al. 2025). These VOs are compatible with established in vitro angiogenesis assays and kidney capsule transplantation, providing a flexible and efficient platform for studying vascular biology.

### Development of the protocol

During development, vascular progenitor cells arise from the lateral plate mesoderm and give rise to both ECs and MCs (Jain 2003; Gaengel et al. 2009; Koyano-Nakagawa and Garry 2017). Guided by this developmental trajectory, most current differentiation protocols rely on the stepwise transition of hPSCs into mesoderm progenitor cells (MePCs), followed by specification into vascular lineages (Lian et al. 2014; Zhang et al. 2019). Typically, activation of Wnt signaling directs hPSCs toward a mesodermal fate, whereas subsequent activation of VEGF and FGF signaling, together with inhibition of TGF-β signaling, promotes EC specification (James et al. 2010). In contrast, activation of TGF-β and PDGF signaling induces an MC fate (Samuel et al. 2013; Orlova et al. 2014). These 2D differentiation strategies for generating ECs and MCs are well established and have been applied to engineer vascular networks (Patsch et al. 2015).

However, conventional differentiation approaches are often limited by variable efficiency, prolonged culture duration and batch-to-batch variability, resulting in heterogeneous cell populations. To overcome these limitations, forward programming strategies have been developed to directly induce target cell identities through transient activation of lineage-defining transcription factors (Ng et al. 2020; Joung et al. 2023; Zhao et al. 2023). In the vascular system, ETV2 acts as a master regulator of endothelial specification, whereas NKX3.1 promotes mesenchymal and mural cell programs (Gong et al. 2022; Ahuja et al. 2024). Transient activation of ETV2 or NKX3.1 in hPSCs or MePCs enables rapid and robust generation of ECs or MCs with reduced lineage ambiguity (Wang et al. 2020; Lee et al. 2024; Luo et al. 2024). Transcription factor delivery can be achieved using a doxycycline (Dox)-inducible system for routine laboratory applications or mRNA transfection for translational purposes (Zhao et al. 2023). ECs and MCs generated by forward programming are referred to here as Dox-ECs and Dox-MCs. Co-transplantation of Dox-ECs and Dox-MCs under the mouse kidney capsule generates functional human vasculature with high MC coverage and perfusion, indicating that forward programming produces functionally competent vascular cells with stable lineage identity after withdrawal of transgene expression (Wang et al. 2020; Lee et al. 2024). Moreover, both human embryonic stem cells (hESCs) and human induced pluripotent stem cells (hiPSCs) are compatible with this approach, with consistent differentiation efficiency, making it suitable for patient-derived iPSC applications with intrinsic heterogeneity.

Based on these findings, we developed a protocol that combines inducible activation of ETV2 and NKX3.1 with 3D organoid culture to generate synthetic vascular organoids (Syn-VOs) containing ECs and MCs. Compared with Chem-VOs generated using growth factor-based differentiation, in which MCs arise as a byproduct, Syn-VOs exhibit more consistent EC proportions and a tunable EC-to-MC ratio. When combined with fluorescent reporter hPSC lines, vascular networks formed in hydrogel or after kidney capsule transplantation can be directly visualized and evaluated. Overall, this protocol simplifies VO differentiation using orthogonal programming and provides a reproducible platform for in vitro and in vivo vascular biology studies.

### Overview of the procedure

This protocol describes experimental procedures for hPSC line establishment, Syn-VO generation, in vitro angiogenesis assays, and in vivo kidney capsule transplantation, followed by instructions for Syn-VO characterization and confocal imaging. First, we detailed the method for electroporation of hPSCs and incorporation of dox-inducible cassettes for transient activation of ETV2 and NKX3.1 (Steps 1–17). Establishing stable hPSC lines facilitates reproducible generation of Syn-VOs for routine experiments. Next, we described the method for generating Syn-VOs via orthogonal programming, as well as the method for generating Chem-VOs via conventional differentiation using growth factors as controls (Steps 18–23). Then, for direct visualization of human vasculature in hydrogel or under the kidney capsule, we developed a method for generating *PECAM1* and *ACTA2* reporter hPSC lines using established donor and sgRNA plasmids (Steps 24–33). Although reporter line generation requires additional genome editing, these lines facilitate rapid assessment of vascular structure and organization, supporting applications such as drug screening and disease modeling. Then, we described the method for embedding the Syn-VOs in a hydrogel for in vitro angiogenesis and direct confocal microscopy imaging (Steps 34–46). This model enables the potential screening of pro-angiogenic or anti-angiogenic factors. Finally, we described the method for kidney capsule transplantation and the method for testing perfusion and imaging of the vascular network (Steps 47–60). Notably, free Syn-VOs are injectable without using any exogenous hydrogel, simplifying the transplantation procedures.

### Applications of the method

#### Drug screening

Syn-VOs can be incorporated into in vitro angiogenesis assays by embedding them in a hydrogel, enabling visualization of vascular sprouting, network formation, and EC-MC interactions. When combined with fluorescent reporter hPSC lines, this system enables real-time assessment of vascular dynamics without additional staining, providing a convenient and scalable platform for screening pro-angiogenic or anti-angiogenic compounds and for evaluating drug toxicity and pathway-specific effects on human vascular networks. Combining high-content imaging with a visualized angiogenesis assay enables high-throughput drug screening using authentic human vasculature (Zhao et al. 2025).

#### Disease modeling

Syn-VO is compatible with disease modeling using patient-derived hiPSCs or genetically engineered hPSCs carrying pathogenic mutations. Syn-VOs generated from these cell lines enable investigation of vascular abnormalities, such as defects in vessel morphology, mural cell coverage, or angiogenic capacity in a human-specific context. This approach is suitable for modeling inherited vascular disorders, such as cerebral autosomal dominant arteriopathy with subcortical infarcts and leukoencephalopathy (CADASIL), cerebral cavernous malformations (CCM), and hereditary hemorrhagic telangiectasia (HHT), as well as vascular malformations driven by somatic mutations such as *TEK* and *PIK3CA*.

#### Co-transplantation

Syn-VOs can be applied in co-transplantation experiments to promote graft vascularization and survival. For instance, co-transplantation of Syn-VOs with mouse primary pancreatic islets under the kidney capsule leads to the formation of functional intra-islet vasculature and improves islet performance when suboptimal islet numbers are transplanted. By facilitating rapid revascularization, Syn-VOs enable effective reversal of hyperglycemia with reduced islet mass in diabetic mouse models (Gong et al. 2025). This co-transplantation strategy may be broadly applicable to other tissues or organoid systems that depend on efficient vascular integration following transplantation.

#### Regenerative medicine

The injectable nature and defined cellular composition of Syn-VOs make them well-suited for regenerative medicine applications. Compared with transplantation of dissociated ECs and MCs, Syn-VOs show improved engraftment in murine hind-limb ischemia models, likely due to their preorganized 3D architecture. Upon transplantation, Syn-VOs efficiently integrate with host tissue, restore perfusion, and reduce ischemia-induced necrosis, highlighting their translational potential (Gong et al. 2025). In addition, the mRNA-based induction strategy enables footprint-free generation of VOs, further supporting their safety for clinical translation.

### Comparison with other methods

Several studies have reported methods for generating vascular networks from hPSCs. A common approach is to utilize the in vivo environment to support vasculogenesis and angiogenesis. Co-transplantation of hPSC-derived ECs together with pericytes or mesenchymal stromal cells (MSCs), either epidurally or under the kidney capsule, can form functional human vasculature (Samuel et al. 2013; Wimmer et al. 2019b; Wang et al. 2020). Further mechanistic studies suggested that host neutrophils facilitate engraftment of the human vascular network, and that mitochondrial transfer supports the early survival of ECs via mitophagy (Lin et al. 2017, 2024). These findings highlight the importance of host-graft interactions and the requirement for stromal cells in this model. This approach enables the formation of human-mouse chimeric blood vessels, in which the human-derived vasculature can be perfused, allowing live imaging as well as histological analysis of vascular structures derived from healthy or patient-derived iPSCs.

The most advanced in vitro model of human vascular networks derived from hPSCs is based on EB differentiation combined with ECM embedding (Wimmer et al. 2019a, 2019b). In this system, hPSCs are first aggregated and differentiated toward mesoderm using CHIR99021 and BMP4, followed by vascular lineage induction with VEGF-A and forskolin. Upon embedding in ECM, the organoids spontaneously sprout and form stable vascular networks covered by pericytes and surrounded by a basement membrane. In addition, individual vascular organoids can be isolated from the ECM and transplanted under the kidney capsule, where human vessels, including arteries, arterioles and venules, can form and become perfused. This method has been applied to study diabetic vasculopathy and led to the identification of key molecular drivers, including DLL4 and NOTCH3, that play important roles in disease pathophysiology (Wimmer et al. 2019b).

While the above methodologies provide useful tools for studying vascular biology, our protocol is characterized by the rapid, robust generation of VOs from hPSCs. By activating transcription factors, the cellular composition of the organoids is more defined and reproducible (Zhao et al. 2023). This forward programming strategy overcomes a major limitation of conventional hPSC differentiation approaches, which is the variability in differentiation efficiency toward vascular lineages. Activation of ETV2 or NKX3.1 robustly induces the generation of ECs or MCs across multiple hESC and hiPSC lines (Wang et al. 2020; Lee et al. 2024). When adapted to organoid culture, this approach enables the proportions of ECs and MCs to be tuned, facilitating more flexible experimental designs, particularly when incorporating VOs to vascularize other tissues. Furthermore, these VOs are compatible with previously established in vitro angiogenesis assays and in vivo transplantation models, allowing this method to be readily adopted by laboratories with these experimental setups.

### Experimental design

#### Genome editing of hPSCs

For routine laboratory applications, we recommend establishing stable cell lines for the inducible activation of ETV2 and NKX3.1. Notably, the coding sequences (CDS) of different isoforms can influence differentiation efficiency (Ng et al. 2020). Thus, the full sequence of ETV2 and NKX3.1 CDS should be checked when cloning for the plasmids. Successful genome editing depends critically on efficient transfection and the survival of hPSCs post-transfection. In this protocol, we detail an electroporation method using the Lonza Nucleofector 2b for plasmid delivery. For first-time hPSC electroporation, it is strongly recommended to test multiple electroporation protocols and cell input, and to include a fluorescent plasmid to assess transfection efficiency. For 2–3 × 10⁶ hPSCs, total DNA should not exceed 4 μg, as higher amounts can cause substantial cell death. A post-electroporation survival rate of at least 20% is required to enable proliferation. At least eight clones should be picked for screening to isolate pure iETV2 or iNKX3.1 clones. The 3 × HA epitope can be used to confirm expression and check clonal purity.

For the generation of *PECAM1* and *ACTA2* reporter lines, we validated the plasmid sets on three hPSC lines. Since the knock-in allele is typically single-copy, drug resistance in edited hPSCs is lower than in dox-inducible lines (which are multi-copy) and may vary across hPSC lines. We recommend starting selection at 0.5 μg/mL puromycin to avoid complete cell death. After the first enrichment, cells can be split into multiple wells for gradient puromycin selection. In our experience, enriched populations can tolerate up to 2.5 μg/mL puromycin, and subsequent clone picking under higher selection pressure facilitates isolation of pure clones.

#### Generation of VOs

The first step in generating VOs is directing hPSCs toward MePCs, which shows minimal variability across hPSC lines. Activation of Wnt signaling with 6 μM CHIR99021 efficiently induces MePCs. Routine quality control is generally not required, although detachment of MePCs at day 1 or 2 may indicate poor cell quality. Next, MePCs are dissociated and combined to simultaneously induce EC and MC lineages via transient activation of ETV2 and NKX3.1. For initial differentiations, we suggest testing EC-to-MC ratios of 1:1, 2:1, and 1:2, followed by flow cytometry analysis. In our hands, the proportion of CD31⁺ ECs ranged precisely around 50%, 67%, and 33% across three hPSC lines. Generally, EC proportions above 30% are sufficient, as variations within this range (30%−70%) do not significantly affect vascular network formation in vitro or in vivo. Effective transgene activation requires > 0.5 μg/mL Dox (final concentration), so proper storage of Dox is critical to avoid degradation. *PECAM1* and *ACTA2* reporter lines can be used to monitor differentiation dynamics: *PECAM1-mRuby3* signal emerges by the end of day 3 and intensifies by day 4, whereas *ACTA2-EGFP* gradually appears from day 4. Routine flow cytometry at day 6 is recommended to assess Syn-VO quality using the standard threshold of > 30% ECs.

#### Formation of vascular network

Syn-VOs can be embedded in a hydrogel or transplanted under the kidney capsule to form functional human vasculature. For in vitro drug evaluation, we recommend a 1–2–2 workflow: 1 day in normal medium to initiate vascularization, followed by 2 days with the factor of interest, and then another 2 days with the factor, for a total of 5 days to assess drug effects. For in vivo transplantation, we suggest using luciferase reporter lines initially to evaluate engraftment efficiency. Once the workflow is established, *PECAM1* and *ACTA2* reporter lines can be used for live imaging. Vascular networks are observable by 7 days post-transplantation and remain stable for at least one month under the kidney capsule. Optimal assessment includes intravenous injection of tomato lectin or dextran to evaluate perfusion, followed by live imaging to visualize human vasculature, and subsequent fixation and sectioning for immunostaining—allowing three complementary analyses from a single mouse.

## Materials

### Biological materials

- H1 hESC (WiCell, cat. no. WA01)
- iPSC-F (Shownin Biotechnologies, cat. no. RC01001-B)
- iPSC18 (Takara, cat. no. Y00305)
  ▲ CAUTION The use of human embryonic stem cells and human induced pluripotent stem cells must comply with all applicable institutional, national and ethical regulations. All required approvals should be obtained before initiating experiments.
- PB-iETV2-3 × HA (Wekwik, cat. no. 0001987)
- PB-iNKX3.1–3 × HA (Wekwik, cat. no. 0002016)
- PECAM1-mRuby3-T2A-secNluc (Addgene, cat. no. 235607; Wekwik, cat. no. 0001096)
- PECAM1-3’gRNA (Addgene, cat. no. 235606; Wekwik, cat. no. 0001097)
- pACTA2_HL-P2A-eGFP-PGK-PuroR-ACTA2_HR (Addgene, cat. no. 126705; Wekwik, cat. no. 0002014)
- pU6-ACTA2_gRNA1-SpCas9-T2A-GFP (Addgene, cat. no. 126706; Wekwik, cat. no. 0002015)

### Reagents

- mTeSR1 medium (StemCell Technologies, cat. no. 05850)
- 0.02% EDTA Solution (Procell, cat. no. PB180320)
- Matrigel hESC-qualified (Corning, cat. no. 354277)
- Matrigel growth factor reduced (Corning, cat. no. 354230)
- PureCol EZ Gel (Advanced Biomatrix, cat. no. 5005)
- Fetal bovine serum (FBS) (Corning, cat. no. 35–108-CV)
- L-Ascorbic acid phosphate (Sigma, cat. no. A8960-5G)
- Penicillin–Streptomycin (P/S) (Procell, cat. no. PB180120)
- TrypLE Express (Thermo Fisher Scientific, cat. no. 12604021)
- Phosphate buffered saline (PBS) (Corning, cat. no. 21–040-CV)
- GlutaMAX (Thermo Fisher Scientific, cat. no. 35050061)
- Advanced DMEM/F12 (Thermo Fisher Scientific, cat. no. 12634028)
- StemPro-34 SFM (Thermo Fisher Scientific, cat. no. 10639011)
- DMEM/F12 (Corning, cat. no. 10–092-CV)
- Doxycycline (Dox) (Topscience, cat. no. T1687)
- Y27632 (Topscience, cat. no. T1870)
- CHIR99021 (Topscience, cat. no. T2310)
- SB431542 (Topscience, cat. no. T1726)
- Puromycin (InvivioGen, cat. no. ant-pr)
- EGF (SinoBiological, cat. no. 10605-HNAE)
- FGF2 (SinoBiological, cat. no. 10014-HNAE)
- VEGF-A (SinoBiological, cat. no. 11066-HNAB)
- EDTA (Thermo Fisher Scientific, cat. no. 15575020)
- Bovine serum albumin (BSA) (Proliant, cat. no. 68700)
- Paraformaldehyde (Sigma, cat. no. 158127)
- EDTA antigen retrieval solution (ZSGB-BIO, cat. no. ZLI-9069)
- Donkey serum (Servicebio, cat. no. G1217)
- Mounting medium with DAPI (Servicebio, cat. no. G1407)
- Lycopersicon Esculentum (Tomato) Lectin, DyLight 649 (Thermo Fisher Scientific, cat. no. L32472)
- Dextran-Cy5, 70,000 Da (Yusi Medicine Technology, cat. no. YS-De1623)
- MojoSort Human anti-PE Nanobeads (Biolegend, cat. no. 480092)

### Antibodies

▲ CRITICAL The usage and dilution factor are listed in Table 1

**Table 1:** Antibodies for immunofluorescence

Antigen	Cat. no	Sample	Dilution	Reactivity
CD31	ab76533	Syn-VO, graft	1:200	Human
CD31	AF806	Dox-EC, Syn/Chem-VO, graft	1:200	Human
PDGFRβ	AF385	Dox-EC, Syn/Chem-VO, graft	1:200	Human, mouse
SOX17	AF1924	Syn/Chem-VO	1:500	Human
HA tag	3724	Edited hPSCs	1:200	N/A
vWF	sc-53466	Dox-EC	1:100	Human
α-SMA	sc-32251	Dox-MC	1:100	Human, mouse
α-SMA	ab5694	Graft	1:200	Human, mouse
Calponin	sc-58707	Dox-MC	1:200	Human, mouse
KU80	2180	Graft	1:200	Human
COL IV	ab214417	Graft	1:200	Human
COUP-TFII	ab211777	Graft	1:200	Human, mouse

- PDGFRβ-PE (BD Biosciences, cat. no. 558821; RRID: AB_397132)
- CD31-APC (Biolegend, cat. no. 303116; RRID: AB_1877151)
- CD31-PE (BD Biosciences, cat. no. 555446; RRID: AB_395839)
- Rabbit anti-human CD31 (Abcam, cat. no. ab76533; RRID: AB_1523298)
- Sheep anti-human CD31 (R&D systems, cat. no. AF806; RRID: AB_355617)
- Goat anti-PDGFRβ (R&D systems, cat. no. AF385; RRID: AB_355339)
- Goat anti-SOX17 (R&D systems, cat. no. AF1924; RRID: AB_355060)
- Rabbit anti-HA tag (Cell Signaling Technology, cat. no. 3724; RRID: AB_1549585)
- Mouse anti-vWF (Santa Cruz Biotechnology, cat. no. sc-53466; RRID: AB_630439)
- Mouse anti-α-SMA (Santa Cruz Biotechnology, cat. no. sc-32251; RRID: AB_262054)
- Rabbit anti-α-SMA (Abcam, cat. no. ab5694; RRID: AB_2223021)
- Mouse anti-Calponin (Santa Cruz Biotechnology, cat. no. sc-58707; RRID: AB_781770)
- Rabbit anti-human KU80 (Cell Signaling Technology, cat. no. 2180; RRID: AB_2218736)
- Rabbit anti-collagen IV (Abcam, cat. no. ab214417; RRID: AB_2801511)
- Rabbit anti-NR2F2 (Abcam, cat. no. ab211777; RRID: AB_2895604)
- Donkey anti-mouse IgG, Alexa Fluor 488 (Thermo Fisher Scientific, cat. no. A21202; RRID: AB_141607)
- Donkey anti-mouse IgG, Alexa Fluor 568 (Thermo Fisher Scientific, cat. no. A11037; RRID: AB_11180865)
- Donkey anti-mouse IgG, Alexa Fluor 647 (Thermo Fisher Scientific, cat. no. A31571; RRID: AB_162542)
- Donkey anti-rabbit IgG, Alexa Fluor 488 (Thermo Fisher Scientific, cat. no. A21206; RRID: AB_2535792)
- Donkey anti-rabbit IgG, Alexa Fluor 568 (Thermo Fisher Scientific, cat. no. A10042; RRID: AB_2534017)
- Donkey anti-rabbit IgG, Alexa Fluor 647 (Thermo Fisher Scientific, cat. no. A31573; RRID: AB_2536183)
- Donkey anti-goat IgG, Alexa Fluor 488 (Thermo Fisher Scientific, cat. no. A11055; RRID: AB_2534102)
- Donkey anti-goat IgG, Alexa Fluor 594 (Thermo Fisher Scientific, cat. no. A11058; RRID: AB_2534105)
- Donkey anti- goat IgG, Alexa Fluor 647 (Thermo Fisher Scientific, cat. no. A21447; RRID: AB_2535864)

### Equipment

- Tissue culture plate, 6-well (Jet Biofil, cat. no. TCP010006)
- Tissue culture plate, 6-well, non-treated (Servicebio, cat. no. CCP-6N)
- Tissue culture plate, 12-well (Jet Biofil, cat. no. TCP010012)
- Tissue culture plate, 24-well (Jet Biofil, cat. no. TCP010024)
- 15 mL centrifuge tube (Labselect, cat. no. CT-002–15)
- 50 mL centrifuge tube (Jet Biofil, cat. no. CFT011500)
- Cryogenic tube (Corning, cat. no. 430659)
- 5 mL serological pipette (Jet Biofil, cat. no. GSP010005)
- 10 mL serological pipette (Jet Biofil, cat. no. GSP010010)
- Insulin syringe, 30G (BD Biosciences)
- 35 mm Glass bottom dishes (Cellvis, cat. no. D35-20–1.5-N)
- Lonza Nucleofector 2b (Lonza, model no. AXP-1001)
- FACSymphony S6 Cell Sorter (BD Biosciences)
- CO_2_ incubator (ESCO, model no. CLM-170B-8)
- Biological safety cabinet (ESCO, model no. AC1-3E8)
- Orbital shaker (Jimei Electronic, model no. CO-06U)
- Fluorescent microscope (Olympus, model no. CKX53)
- Confocal microscope (Nikon, model no. AXR)

### Reagent setup

#### Maintenance of feeder-free hPSCs

We maintain hPSC in mTeSR1 medium on Matrigel-coated plates. Briefly, dissolve 1 mL hESC-qualified Matrigel in 500 mL DMEM/F12 (1:500). Add 1 mL diluted Matrigel solution in one well of a six-well plate and put the plate in a 37 °C CO_2_ incubator for at least 30 min. Routinely passage hPSCs using 0.02% EDTA (0.5 mM) and split at a 1:6 ratio. Cells will reach near confluency after 3 days. After thawing, hPSCs should be passaged at least once before differentiation towards ECs or MCs.

#### L-Ascorbic acid phosphate stock solution (30 mg/mL)

Dissolve 0.6 g L-Ascorbic acid phosphate in 20 mL ddH_2_O. Sterile-filter the solution using a 0.22-μm filter. Divide the solution into 1 mL aliquots under sterile conditions. Store the aliquots at −20 °C for up to 1 year.

#### CHIR99021 stock solution (20 mM)

Dissolve 10 mg CHIR99021 (MW: 465.34 g/mol) in 1.075 mL DMSO under sterile conditions. Divide the solution into 20 μL aliquots and store the aliquots at −20 °C for up to 6 months.

▲ CRITICAL Avoid repeated freeze–thaw cycles of CHIR99021. Aliquots should be discarded after more than three freeze–thaw cycles.

#### Dox stock solution (0.5 mg/mL)

Dissolve 10 mg Dox in 20 mL ddH_2_O and sterile-filter the solution using a 0.22-μm filter. Divide the solution into 20 μL aliquots under sterile conditions. Store the aliquots at −20 °C for up to 1 year.

#### Growth factor stock solution (100 ng/μL)

For preparation of EGF, FGF2 and VEGF-A stock solution, dissolve 1 mg protein in 10 mL DMEM/F12 medium under sterile conditions. Divide the solution into 250 μL aliquots for FGF2 and VEGF-A and 50 μL aliquots for EGF for preparation of 500 mL S2 medium. Store the aliquots at −80 °C for up to 1 year.

#### Basal medium

Mix 500 mL Advanced DMEM/F12, 5 mL GlutaMAX (100 ×, final concentration 1 ×), 1 mL P/S (100 ×, final concentration 0.2 ×), 1 mL L-Ascorbic acid phosphate (30 mg/mL, final concentration 60 μg/mL). Store at 4 °C for up to 1 month.

#### S1 medium

Mix 10 mL basal medium and 3 μL CHIR99021 (20 mM, final concentration 6 μM). Prepare fresh for use and do not store this medium.

#### S2 medium

Mix 500 mL basal medium, 50 μL EGF (100 ng/μL, final concentration 10 ng/mL), 250 μL FGF2 (100 ng/μL, final concentration 50 ng/mL), 250 μL VEGF-A (100 ng/μL, final concentration 50 ng/mL). Store at 4 °C for up to 1 month.

#### MC medium

Mix 49 mL basal medium, 1 mL FBS (2% v/v), 5 μL FGF2 (100 ng/μL, final concentration 10 ng/mL), 2.5 μL EGF (100 ng/μL, final concentration 5 ng/mL). Store at 4 °C for up to 1 month.

#### Angiogenesis medium

Mix 42.5 mL StemPro-34 SFM complete medium, 7.5 mL FBS (15% v/v), 50 μL FGF2 (100 ng/μL, final concentration 100 ng/mL), 50 μL VEGF-A (100 ng/μL, final concentration 100 ng/mL). Store at 4 °C for up to 1 month.

#### Hydrogel solution

Mix 3 mL PureCol EZ Gel (Collagen I solution) with 1 mL growth-factor reduced Matrigel. Prepare fresh and do not store this solution.

#### Paraformaldehyde solution (4% w/v)

Dissolve 20 g paraformaldehyde in 400 mL PBS under constant stirring at 55–60 °C in a fume hood. Add 1–2 drops of 1 M NaOH to facilitate dissolution until the solution becomes clear. Allow the solution to cool to room temperature (RT), and bring the final volume to 500 mL with PBS. Filter the solution through a 0.22 μm filter, and store at 4 °C for up to 1 month.

▲ CAUTION Paraformaldehyde is toxic. Prepare the solution in a fume hood while wearing appropriate personal protective equipment. Avoid inhalation of vapors and skin contact, and appropriately dispose of waste.

#### Blocking buffer

Prepare a 5% v/v donkey serum blocking buffer by diluting 2 mL donkey serum in 38 mL PBS. Filter the solution through a 0.22 μm filter and store the buffer at 4 °C for up to 2 months.

#### FACS buffer

Prepare FACS buffer by dissolving 0.2 g BSA in 39.6 mL PBS, then adding 400 μL of 0.5 M EDTA. Mix thoroughly until fully dissolved, filter through a 0.22 μm filter, and store at 4 °C for up to 2 months.

## Procedure

### Generation of iETV2 and iNKX3.1 hPSC lines

● Timing ~ 1 month

▲ CRITICAL We maintain hPSCs under feeder-free conditions in mTeSR1 medium on Matrigel and passage the cells every 3 days using EDTA. Proper maintenance of hPSCs is essential for successful downstream differentiation. For long-term maintenance of iETV2 and iNKX3.1 hPSC lines, we closely monitor spontaneous differentiation.

1. Culture wild-type hPSCs under standard conditions. Before electroporation, passage the cells at least once after thawing to ensure recovery.
2. Introduce either the PB-iETV2 (Wekwik #0001987) or PB-iNKX3.1 (Wekwik #0002016) donor plasmid together with a piggyBac transposase–expressing plasmid (Wekwik #0001992) by electroporation to establish stable hPSC lines. Use cells from one near-confluent well of a six-well plate for each electroporation.
3. Wash the hPSCs once with PBS. Add 1 mL EDTA per well and incubate at 37 °C for 6–7 min.
4. Gently triturate the cells, transfer them to a 15-mL centrifuge tube and centrifuge at 300* g* for 3 min at RT to pellet the cells. Aspirate the supernatant and resuspend the cell pellet in 100 μL mTeSR1.
5. Mix the cell suspension with 2 μg donor plasmid and 1 μg transposase plasmid, and transfer the entire mixture to an electroporation cuvette. Electroporate the cells using program “B-016” on a Lonza Nucleofector 2b device.
  ▲ CRITICAL While this protocol is optimized for the Lonza Nucleofector 2b, other systems may be used. For the Neon Transfection System (Invitrogen), use the following parameters: 1,150 V (pulse voltage), 30 ms (pulse width), and 2 pulses. If utilizing other electroporation platforms, refer to the manufacturer’s guidelines for cell-type-specific optimization to ensure maximum viability and efficiency.
6. Immediately transfer the electroporated cells into one Matrigel-coated well of a six-well plate pre-filled with 2 mL mTeSR1 supplemented with 5 μM Y27632, and return the plate to the incubator.
  ▲ CRITICAL STEP Proliferative and healthy hPSCs survive well after electroporation and typically reach near confluency within 48 h after electroporation.
  ♦ TROUBLESHOOTING
7. At 48 h post electroporation, initiate drug selection by replacing the medium with mTeSR1 supplemented with 5 μg/mL puromycin.
  ▲ CRITICAL STEP If the cells reach confluency within 24 h after electroporation, passage them at a 1:3 ratio and initiate the first round of drug selection once confluency is reached again.
  ♦ TROUBLESHOOTING
8. After 24 h of selection, withdraw puromycin and replace the medium with fresh mTeSR1. When the cells reach confluency again, perform a second round of selection using 10 μg/mL puromycin.
9. Upon reaching confluency, passage the cells once to allow recovery. Initiate clonal isolation by dissociating the cells into single cells using TrypLE and plating them into a 10-cm dish.
10. Wash the cells once with PBS, add 1 mL TrypLE per well and incubate at 37 °C for 3 min.
11. Gently tap the plate to facilitate dissociation, neutralize TrypLE with 1 mL DMEM/F12 and pipette 3–5 times. Collect the cells into a 15-mL centrifuge tube and centrifuge at 300*g* for 3 min at RT.
12. Aspirate the supernatant and resuspend the cells in 1 mL mTeSR1 supplemented with 5 μM Y27632. Count the cells using a hemocytometer or automated cell counter. Plate approximately 3,000 cells into a Matrigel-coated 10-cm dish containing 8 mL mTeSR1 supplemented with 5 μM Y27632.
13. Replace the medium completely with 8 mL fresh mTeSR1 at 3 days and 6 days post seeding. Pick individual clones between day 7 and 10.
14. Under an inverted microscope, manually pick individual clones by gently scraping with a P200 pipette and transfer each clone into one well of a prepared 24-well plate. Pipette three times to break the clone into small fragments.
15. Replace 500 μL mTeSR1 every other day until 6 days after clone picking.
16. Passage the clones into a 12-well plate using EDTA, followed by simultaneous passaging into a 6-well plate and a 12-well plate. Treat cells in the 12-well plate with Dox for 24 h and perform immunostaining to assess clone purity.
17. Select the optimal clone for downstream applications. Verify differentiation potential by inducing the iETV2 cell line toward Dox-EC (Option A) or the iNKX3.1 cell line toward Dox-MC (Option B).
  ▲ CRITICAL STEP When selecting stable single-cell clones, ensuring a high HA-tag positive rate (>99%) is necessary but not sufficient. It is essential to evaluate the differentiation efficiency and transgene expression levels of ETV2 or NKX3.1 upon Dox induction. It is highly recommended to select 2–3 clones with high expression levels, as insufficient expression may compromise differentiation efficiency and yield heterogeneous cell populations.
  (A) Dox-EC induction.
    (i) On d-1, passage iETV2 hPSCs into one well of a 12-well plate at a ratio of 1:6.
    (ii) On d0 and d1, replace the medium with 1 mL S1 medium for 2 days to generate MePCs.
    (iii) On d2, passage MePCs onto coverslips in a 12-well plate using EDTA at a 1:12 ratio in 1 mL basal medium supplemented with 0.5 μg/mL Dox.
    (iv) On d3, replace the medium with 1 mL S2 medium.
    (v) At the end of d3, fix the cells with 4% PFA and perform immunostaining for EC markers CD31 and vWF.
  (B) Dox-MC induction.
    (i) On d-1, passage iETV2 hPSCs into one well of a 12-well plate at a ratio of 1:6.
    (ii) On d0 and d1, replace the medium with 1 mL S1 medium for 2 days to generate MePCs.
    (iii) On d2, passage MePCs onto coverslips in a 12-well plate using EDTA at a 1:10 ratio in 1 mL basal medium supplemented with 0.5 μg/mL Dox.
    (iv) On d3, replace the medium with 1 mL basal medium supplemented with 0.5 μg/mL Dox.
    (v) On d4 and d5, replace the medium with 1 mL MC medium.
    (vi) At the end of d5, fix the cells with 4% PFA and perform immunostaining for MC markers α-SMA and calponin.

■ PAUSE POINT Established iETV2 and iNKX3.1 hPSC lines can be expanded and cryopreserved for future use.

▲ CRITICAL If clonal purity does not reach 100%, include 10 μg/mL puromycin for the first 24 h after passaging in at least one of the two passages for maintenance.

### Generation of vascular organoids via activation of ETV2 and NKX3.1

● Timing 6 d

18. On d-1, passage iETV2 hPSCs and iNKX3.1 hPSCs into a 6-well plate at a 1:6 ratio.
  ▲CRITICAL STEP Cells should reach ~30–40% confluency at 24 h post passaging. For accurate seeding, plate 5 × 10^5^ cells per well (approximately 52,000 cells/cm^2^).
19. On d0, replace the medium with 2 mL S1 medium per well for 2 days to generate MePCs.
  ♦ TROUBLESHOOTING
20. On d2, wash the cells once with PBS. Add 1 mL TrypLE per well and incubate at 37 °C for 1 min to dissociate MePCs.
21. Gently tap the plate to facilitate dissociation, neutralize TrypLE with 1 mL DMEM/F12, and pipette 3–5 times. Collect the cell suspension into a 15-mL centrifuge tube and centrifuge at 300* g* for 3 min at RT.
  ▲ CRITICAL STEP MePCs typically reach near confluency and dissociate readily into single cells. Avoid prolonged TrypLE incubation to optimize cell viability.
22. Resuspend the cells in basal medium and pass the suspension through a 40-μm cell strainer to remove cell aggregates. Count the cells using a hemocytometer or an automated cell counter. Generate Syn-VOs by activating ETV2 and NKX3.1 (Option A, Table 2). In parallel, generate Chem-VOs as a control (Option B).
  (A) Generation of Syn-VO.
    (i) Combine 2 × 10⁶ iETV2 MePCs and 2 × 10⁶ iNKX3.1 MePCs in 4 mL basal medium supplemented with 0.5 μg/mL Dox. Plate the mixture into one well of a non-treated six-well plate and place the plate on an orbital shaker in the incubator at 100 rpm.
      ▲ CRITICAL STEP Alternative endothelial-to-mural cell ratios can be achieved by adjusting the relative proportions of MePCs derived from the two lines. The rotation speed may need to be optimized.
    (ii) On d3, collect the vascular organoids into a 15-mL or 50-mL centrifuge tube, depending on volume, and centrifuge at 100* g* for 1 min at RT to pellet the organoids. Aspirate the supernatant, resuspend the organoids in 4 mL S2 medium, and return the suspension to the original well. Place the plate back on the orbital shaker.
    (iii) On d4 and d5, no need to change the medium. Syn-VO is generated at the end of d6 and can be used for downstream applications.
      ▲ CRITICAL STEP Prolonged culture beyond d6 is not recommended, as the proportion of ECs gradually decreases with extended culture.
      ♦ TROUBLESHOOTING
  (B) Generation of Chem-VO.
    (i) On d2, plate 4 × 10⁶ MePCs derived from either iETV2 or iNKX3.1 hPSCs in 4 mL S2 medium supplemented with 10 μM SB431542 into one well of a non-treated six-well plate. Place the plate on an orbital shaker in the incubator at 100 rpm.
    (ii) On d3, d4 and d5, replace half of the medium with fresh S2 medium supplemented with 10 μM SB431542. Chem-VOs are generated by the end of d6 and can be used as control samples.

**Table 2:** Syn-VO differentiation schedule

Stage	Day	Medium	Factor	Final conc	Dilution
S0	d-1	mTeSR1	Y27632	5 μM	1:1000
S1	d0-d1	Basal medium	CHIR99021	6 μM	1:3333
S2	d2	Basal medium	Dox	0.5 μg/mL	1:1000
	d3-d5	Basal medium	VEGF-A	50 ng/mL	1:2000
			FGF2	50 ng/mL	1:2000
			EGF	10 ng/mL	1:10,000
23. Characterize the generated vascular organoids by immunostaining (Option A) or flow cytometry (Option B) or RT-qPCR (Option C).
  (A) Histology analysis for vascular organoids.
    (i) To perform immunostaining on VOs after 3D culture, the organoids need to be embedded in paraffin and sectioned into slides. To begin this procedure, collect VOs using a P1000 pipette and transfer them into a 1.5-mL microcentrifuge tube. Centrifuge at 100* g* for 1 min at RT to pellet the organoids.
      ▲ CRITICAL STEP Collect a sufficient number of vascular organoids (typically more than one-quarter of VOs in a well) to ensure that multiple organoids are present in each paraffin section.
    (ii) Aspirate the supernatant and wash the organoids once with 1 mL PBS.
    (iii) Fix the organoids by adding 500 μL 4% PFA and incubating for 30 min at RT.
    (iv) Centrifuge at 100* g* for 1 min at RT to pellet the VOs. Remove and properly dispose PFA. Wash the VOs once with 1 mL PBS.
    (v) Remove the PBS and resuspend the organoids in ~ 20 μL pre-warmed low melting point agarose. Allow the agarose to solidify at 4 °C for 30 min.
    (vi) Process the agarose-embedded organoids for standard paraffin embedding and section them onto glass slides.
    (vii) Deparaffinize sections by incubating slides twice in xylene for 5 min each.
    (viii) Rehydrate sections through a graded ethanol series (100%, 95%, 80% and 70%) for 3 min each, followed by a final rinse in distilled water.
    (ix) Perform antigen retrieval by heating the slides in EDTA buffer (1 mM EDTA, pH 9.0) at 95–100 °C for 20 min.
      ▲ CRITICAL STEP The CD31 and PDGFRβ antibodies used in this study are also compatible with antigen retrieval using citrate buffer (10 mM sodium citrate, pH 6.0).
    (x) Allow the slides to cool to RT and wash three times with PBS, 5 min per wash.
    (xi) Block the sample in blocking buffer (5% donkey serum in PBS) for 1 h at RT.
    (xii) Incubate sections with antibodies against CD31 and PDGFRβ diluted in blocking buffer overnight at 4 °C in a humidified chamber.
    (xiii) Wash sections three times with PBS, 5 min per wash.
    (xiv) Incubate sections with appropriate fluorophore-conjugated secondary antibodies diluted in blocking buffer for 1 h at RT in the dark.
    (xv) Wash sections three times with PBS, 5 min per wash.
    (xvi) Mount sections with antifade mounting medium containing DAPI and apply coverslips, protecting the slides from light.
    (xvii) Seal the coverslips using a sealing solution such as clear nail polish. Store slides at 4 °C until imaging by confocal or wide-field fluorescence microscopy.
  (B) Flow cytometry analysis for vascular organoids.
    (i) To perform flow cytometry analysis on VOs, dissociate VOs into single cells using TrypLE. Collect vascular organoids and centrifuge at 100 g for 1 min at RT to pellet them.
    (ii) Aspirate the supernatant and wash the organoids once with 5 mL PBS.
    (iii) Resuspend the organoids in 500 μL TrypLE, transfer them into one well of a 12-well plate and place the plate on an orbital shaker in the incubator.
    (iv) After 5 min, gently triturate the suspension 5–10 times and monitor dissociation under a microscope. Once most cells are single cells, neutralize TrypLE with 500 μL DMEM/F12, pass the suspension through a 40-μm cell strainer and transfer the suspension into a 1.5-mL microcentrifuge tube.
    (v) Centrifuge at 300* g* for 3 min at RT to pellet the cells. Fix the cells by adding 500 μL 4% PFA and incubating for 10 min at RT.
    (vi) Centrifuge at 300* g* for 1 min at RT, remove and properly dispose of the PFA and wash the cells once with 1 mL FACS buffer.
    (vii) Dilute FACS antibodies (PDGFRβ-PE and CD31-APC) at 1:100 in FACS buffer. Resuspend the cells in the antibody solution and incubate for 10 min at 4 °C.
    (viii) Centrifuge and remove the antibodies. Wash the cells three times with FACS buffer, 1 mL per wash.
    (ix) Resuspend the cells with FACS buffer and perform analysis using a flow cytometer.
  (C) RT-qPCR analysis for vascular organoids.
    (i) To perform RT-qPCR analysis on VOs, dissociate VOs into single cells using TrypLE as described in Option (B).
    (ii) Perform cell counting and resuspend the cells with sterile FACS buffer (1 × 10^6^ cells/50 μL).
    (iii) Add CD31-PE antibody (1:100) to the cell suspension and mix well. Incubate for 10 min at 4 °C.
    (iv) Centrifuge and remove the antibodies. Wash the cells once with 10 mL FACS buffer.
    (v) Resuspend the cells with sterile FACS buffer (1 × 10^6^ cells/50 μL). Add anti-PE nanobeads (10 μL beads/500 μL buffer) and mix well. Incubate on a rotator for 15 min at 4 °C.
    (vi) Centrifuge and remove the unbound beads. Wash the cells once with 10 mL FACS buffer.
    (vii) Resuspend the cells with 2 mL sterile FACS buffer. Place the tube in the magnet for 2 min to allow CD31^+^ ECs to adhere to the tube wall. Pour out and collect the unlabeled fraction containing CD31^−^ MCs. Repeat this step twice.
    (viii) Centrifuge both fractions to pellet ECs and MCs. Extract total RNA and perform RT-qPCR analysis using standard laboratory protocols. The primer pairs for qPCR analysis are listed in Table 3.

**Table 3:** Primer pairs for qPCR analysis

Gene	Forward primer (5’−3’)	Reverse primer (5’−3’)
*PECAM1*	AACAGTGTTGACATGAAGAGCC	TGTAAAACAGCACGTCATCCTT
*CDH5*	TTGGAACCAGATGCACATTGAT	TCTTGCGACTCACGCTTGAC
*VWF*	CCGATGCAGCCTTTTCGGA	TCCCCAAGATACACGGAGAGG
*ACTA2*	AAAAGACAGCTACGTGGGTGA	GCCATGTTCTATCGGGTACTTC
*TAGLN*	AGTGCAGTCCAAAATCGAGAAG	CTTGCTCAGAATCACGCCAT
*CNN1*	CTGTCAGCCGAGGTTAAGAAC	GAGGCCGTCCATGAAGTTGTT
*SOX17*	GTGGACCGCACGGAATTTG	GGAGATTCACACCGGAGTCA
*EFNB2*	TATGCAGAACTGCGATTTCCAA	TGGGTATAGTACCAGTCCTTGTC
*CXCR4*	ACTACACCGAGGAAATGGGCT	CCCACAATGCCAGTTAAGAAGA
*NR2F2*	TCATGGGTATCGAGAACATTTGC	TTCAACACAAACAGCTCGCTC
*NT5E*	GCCTGGGAGCTTACGATTTTG	TAGTGCCCTGGTACTGGTCG
*EPHB4*	CGCACCTACGAAGTGTGTGA	GTCCGCATCGCTCTCATAGTA
*PROX1*	AAAGGACGGTAGGGACAGCAT	CCTTGGGGATTCATGGCACTAA
*LYVE1*	AGGCTCTTTGCGTGCAGAA	GGTTCGCCTTTTTGCTCACAA
*PDPN*	AACCAGCGAAGACCGCTATAA	CGAATGCCTGTTACACTGTTGA

### Generation of *PECAM1-mRuby3 *and* ACTA2-EGFP* hPSC lines

● Timing 1–2 months

▲ CRITICAL Visualization of vascular network formation in organoid-based in vitro angiogenesis models and in vivo transplantation experiments often relies on tissue clearing and whole-mount immunostaining, which can be technically demanding and time-consuming. To enable rapid and direct visualization of vascular structures, we combine fluorescent reporter hPSC lines with the Syn-VO system. This strategy allows real-time monitoring of EC and MC organization and facilitates the assessment of vascular network formation without additional clearing or staining steps. Here, we describe the generation and application of previously published and validated *PECAM1* and *ACTA2* reporter systems to establish tractable and reproducible in vitro and in vivo angiogenesis assays.

24. Introduce the *PECAM1* or *ACTA2* donor plasmid together with the relative sgRNA plasmid by electroporation, as described above. Use the *PECAM1* donor plasmid (Addgene #235,607; Wekwik #0001096) with *PECAM1* sgRNA plasmid (Addgene #235,606; Wekwik #0001097), or the *ACTA2* donor plasmid (Addgene #126,705; Wekwik #0002014) with *ACTA2* sgRNA plasmid (Addgene #126,706; Wekwik #0002015). Use 2 μg donor plasmid and 1 μg sgRNA plasmid per electroporation.
25. At 48 h post electroporation, initiate drug selection by replacing the medium with mTeSR1 supplemented with 0.5 μg/mL puromycin.
26. After 24 h of selection, withdraw puromycin and replace the medium with fresh mTeSR1. When the cells reach confluency again, perform a second round of selection using 1 μg/mL puromycin.
  ▲ CRITICAL STEP Repeat the selection cycle until no obvious cell death is observed, indicating enrichment of puromycin-resistant cells.
27. For ACTA2-targeted editing, proceed directly to single-cell cloning as described above. For PECAM1-targeted editing, perform FACS to isolate EGFP-negative cells before single-cell cloning to eliminate randomly integrated cells.
  ▲ CRITICAL STEP Sort EGFP-negative cells by FACS before single-cell cloning to enrich for correctly targeted *PECAM1* knockin cells. The donor plasmid contains an EGFP expression cassette outside the homology arms. Cells with random integration of donor plasmids constitutively express EGFP and can thus be excluded.
28. Expand individual clones to six-well plates and extract genomic DNA using a standard protocol. Perform junction PCR for genotyping using the primer pairs listed in Table 4.

**Table 4:** Genotyping primer pairs

Construct	Forward primer (5’−3’)	Reverse primer (5’−3’)	Product size (bp)
PECAM1-mRuby3	GGGTGCTTGATGAAGTTACTTGG	ACTGATGGCCGTTCACGC	774
ACTA2-EGFP	TCTCTGTGCCTAAACCATTCATT	GAAGTTAGTGGCTCCGCTG	803
29. Analyze PCR products by agarose gel electrophoresis to identify correctly sized bands. Excise the appropriate bands, purify the DNA and perform Sanger sequencing to confirm precise knock-in at the target locus.
  ♦ TROUBLESHOOTING
30. Excise the puromycin selection cassette by electroporation of Cre mRNA.
  ■ PAUSE POINT Correctly targeted *PECAM1-mRuby3* and *ACTA2-EGFP* hPSC lines can be expanded and cryopreserved for future use.
31. Functionally validate the reporter hPSC lines by generating Chem-VOs as described above. At the end of d6, assess reporter fluorescence to confirm lineage-specific reporter activation.
32. Introduce the iETV2 cassette into the *PECAM1-mRuby3* reporter hPSC line and the iNKX3.1 cassette into the *ACTA2-EGFP* reporter hPSC line using the procedures described above.
33. Validate the reporter and inducible lines by differentiating *PECAM1-mRuby3/iETV2* cells into Dox-ECs and *ACTA2-EGFP/iNKX3.1* cells into Dox-MCs, as described in Step 17. Perform immunostaining for CD31 or α-SMA, respectively, to confirm concordance between reporter expression and endogenous marker expression.
  ▲ CRITICAL Before expanding clones after two rounds of gene editing, verify that hPSCs maintain a normal karyotype and are free of mycoplasma contamination to ensure cell line integrity and reliability.

### Visualized angiogenesis assay

● Timing 6 d

34. Syn-VO is compatible with the previously reported in vitro angiogenesis model embedded in hydrogel solution. Prepare the hydrogel solution consisting of collagen I and Matrigel mixed at a 3:1 (v/v) ratio. Use reporter hPSC line-derived Syn-VOs to enable direct visualization of vascular network formation by confocal microscopy. For non-reporter Syn-VOs, perform immunostaining according to previously published protocols.
35. Add 100 μL hydrogel solution to each well of a 48-well plate and gently swirl the plate to evenly distribute the solution across the bottom of each well.
  ▲ CRITICAL Keep the hydrogel solution on ice at all times to prevent premature gelation. Larger-format plates (12-well and 24-well) can be used when more vascular networks need to be analyzed, whereas smaller plates facilitate testing multiple conditions within a single batch to minimize batch effects.
36. Incubate the plate at 37 °C for 2 h to allow complete gelation.
37. Under a microscope, manually pick approximately twenty Syn-VOs for one well of a 48-well plate using a P20 pipette and transfer them into a 1.5-mL microcentrifuge tube.
  ▲ CRITICAL STEP Distribute Syn-VOs into multiple 1.5-mL tubes rather than pooling them into a single 15-mL tube to facilitate homogeneous mixing with the hydrogel.
38. Resuspend the Syn-VOs in 100 μL hydrogel solution and gently layer the mixture onto the pre-solidified gel in each well.
39. Incubate the plate at 37 °C for 2 h to ensure complete solidification of the upper hydrogel layer.
40. Add 200 μL prewarmed angiogenesis medium (StemPro-34 SFM supplemented with 15% FBS, 100 ng/mL VEGF-A, and 100 ng/mL FGF2) to each well and return the plate to the incubator. Designate this time point as d1.
41. On d2, small molecules or growth factors can be included to assess the effect on angiogenesis. Here, we use VEGF inhibitor SU5416 (5 μM) and HGF (100 ng/mL) as anti-angiogenic and pro-angiogenic factors. Replace the medium with 200 μL fresh medium.
42. On d4, replace the medium with 200 μL fresh medium.
43. At the end of d5, aspirate the medium and fix the vascular network with 200 μL 4% PFA for 20 min at RT.
  ▲ CRITICAL The culture period can be extended up to d10 to allow formation of larger vascular networks, with medium replaced every other day.
  ♦ TROUBLESHOOTING
44. Remove and properly dispose PFA. Add 300 μL PBS per well. Store the plate at 4 °C.
  ■ PAUSE POINT Fixed reporter hPSC line-derived vascular networks can be stored in PBS at 4 °C for at least 1 week while maintaining robust fluorescent signals.
45. For confocal imaging, gently transfer the hydrogel containing the vascular network to a 35-mm glass-bottom confocal dish using tweezers.
  ▲ CRITICAL STEP Lift the gel from the edge to avoid disrupting vascular structures located near the periphery.
46. Use a 10 × objective to obtain overview images of vascular networks. Use a 20 × objective to visualize EC-MC interactions. Acquire z-stacks with a z-step size that satisfies Nyquist sampling requirements.
  ▲ CRITICAL This visualized assay enables rapid assessment of vascular network formation without removal of the hydrogel. However, oil-immersion objectives cannot be used because the working distance prevents the objective from accessing vascular structures embedded within the gel. In most applications, 10× and 20× objectives provide sufficient resolution for imaging.

### Transplantation of vascular organoids

● Timing 14–28 d

47. Use 8-week-old immunodeficient mice (e.g., BALB/c nude or NSG) for transplantation. Prepare Syn-VOs at the end of d6 of differentiation. For each mouse, use Syn-VOs derived from 8 × 10⁶ MePCs (approximately 1,000 Syn-VOs).
48. Collect Syn-VOs and centrifuge at 100* g* for 1 min at RT. Carefully remove the supernatant and resuspend the organoids in 50–100 μL sterile PBS per mouse.
  ▲ CRITICAL STEP Use the minimal volume required to ensure stable placement of the graft and prevent leakage during injection.
49. Load the Syn-VO suspension into a sterile insulin syringe. Avoid air bubbles.
50. Set up a sterile surgical field and anesthetize the mouse using isoflurane.
51. Place the mouse in a dorsal position. Localize the kidney through the skin of the mouse using fingers.
52. Disinfect the surgical area with iodine tincture followed by 70% ethanol.
53. Make a ~ 1.5 cm incision through the skin and muscle layer to expose the kidney. Squeeze through the kidney and wet the surface of the exposed kidney with normal saline. Repeat this step every few minutes or as needed to prevent the kidney capsule from drying out.
54. Insert the needle shallowly into the renal parenchyma near the lateral border of kidney, advancing parallel to the kidney surface rather than perpendicular to it.
55. Gently advance the needle tip through the parenchyma until it exits into the subcapsular space. Slowly inject the Syn-VO suspension to form a localized graft beneath the renal capsule.
56. Withdraw the needle carefully and press the injection site with a sterile cotton swab for ~ 3 min to prevent leakage and bleeding.
  ♦ TROUBLESHOOTING
57. Gently push the kidney back into the peritoneal cavity.
58. Close the incision by applying sutures to the peritoneal wall and the dermal layer.
59. Place the mouse on a heating pad until full recovery from anesthesia. To minimize postoperative pain, administer an appropriate analgesic. Then return it to its cage for routine monitoring.
60. Analyze grafts 2 or 4 weeks post transplantation. For reporter hPSC line-derived Syn-VOs, perform live imaging (Option A), perfusion assessment (Option B), or histological analysis (Option C).
  ♦ TROUBLESHOOTING
  ▲ CRITICAL A transplantation window of 2–4 weeks is optimal for Syn-VOs to establish functional vascular networks under the kidney capsule. Extending this period further may lead to the overgrowth of MCs or increase the risk of teratoma formation.
  (A) Live imaging of the graft.
    (i) Harvest the graft-containing kidney. Place the kidney into ice-cold normal saline. Immediately perform confocal imaging.
      ▲ CRITICAL STEP *Ex vivo* grafts can be imaged with stable fluorescent signal for at least 3 h after harvest when kept in ice-cold saline. Fixation with PFA markedly reduces fluorescence and is therefore not recommended for live imaging-based analyses.
    (ii) Transfer the kidney to a 35-mm glass-bottom confocal dish with the graft side positioned downward.
    (iii) Acquire overview images of vascular networks using a 10 × objective and higher-resolution images of EC-MC interactions using a 20 × objective. Oil-immersion objectives can also be used.
  (B) Assessment of vascular perfusion.
    (i) To evaluate perfusion of human vessels, intravenously inject fluorescent-labeled tomato lectin (1 mg per mouse) or fluorescent-labeled dextran (1 mg per mouse) 30 min before euthanasia.
    (ii) Harvest the graft-containing kidney and place it in ice-cold normal saline. Immediately perform confocal imaging.
    (iii) Perform confocal imaging using the 568 nm channel to visualize vascular structures and the 647 nm channel to detect perfused vessels.
  (C) Immunostaining of the graft.
    (i) Fix the graft-containing kidney with 4% PFA at RT for at least 2 h.
    (ii) Process the tissue for standard paraffin embedding and section it onto glass slides.
    (iii) Perform immunostaining as described in Step 23. We use antibodies against human CD31 (hCD31) and human KU80 (hKU80) to identify human vessels, and antibodies against hCD31, α-SMA and type IV collagen to assess vascular organization and mural cell coverage.

## Troubleshooting

Troubleshooting advice can be found in Table 5.

**Table 5:** Troubleshooting

Step	Problem	Possible reason	Solution
6	Cells show poor survival after electroporation	Cells are unhealthy	Ensure hPSCs are proliferative and healthy before electroporation
		Incompatible electroporation program	Test alternative electroporation programs according to the manufacturer’s instructions
7	No cells survive after the first round of selection	Low electroporation efficiency	Use GFP- or RFP-expressing plasmids to evaluate transfection efficiency. Test alternative programs to balance efficiency and cell survival
		hPSCs are sensitive to puromycin	Reduce the puromycin concentration to 1 μg/mL
19	MePCs detach from the plate	Poor hPSC quality	Ensure hPSCs are healthy at the start of differentiation
		Medium is too cold	Warm S1 medium to RT before medium change
		MePCs are too dense	Reduce the seeding density of hPSCs at d0
22A	Low proportion of ECs	Inaccurate cell counting	Repeat the experiment with careful cell counting
		Inefficient ETV2 activation by Dox	Check the quality of Dox. Increase the concentration of Dox to 1 μg/mL
		Prolonged culture of Syn-VOs	Avoid maintaining Syn-VOs beyond d6. If extended culture is required, try to include 10 μM SB431542
29	No knock-in clones obtained	Inefficient enrichment during drug selection	Confirm that cells can stably proliferate under 1 μg/mL puromycin
		Poor plasmid quality	Use freshly prepared, high-quality plasmid DNA for genome editing
43	Poor sprouting	Low proportion of ECs	Refer to troubleshooting for Step 22A
		Degradation of growth factors	Confirm the activity of VEGF-A and FGF2
	Generation of very large aggregates	Use too much Syn-VOs	Reduce the number of Syn-VOs per well
		Fusion of multiple aggregates	Improve aggregate distribution during the embedding step
56	Leakage of Syn-VOs	Excessive injection volume	Use no more than 100 μL per mouse
		Insufficient compression time	Maintain pressure at the injection site for at least 3 min after transplantation
	Bleeding	Needle inserted too deeply	Avoid deep penetration to prevent damage to renal blood vessels
60	No detectable graft	Insufficient number of Syn-VOs transplanted	Increase the number of Syn-VOs per mouse
		Poor engraftment of Syn-VOs	Consider using a luciferase reporter line to monitor graft engraftment
	Teratoma formation	Inefficient differentiation	Check the quality of Dox. Perform flow cytometry analysis for quality control

## Results

**Fig. 1:**
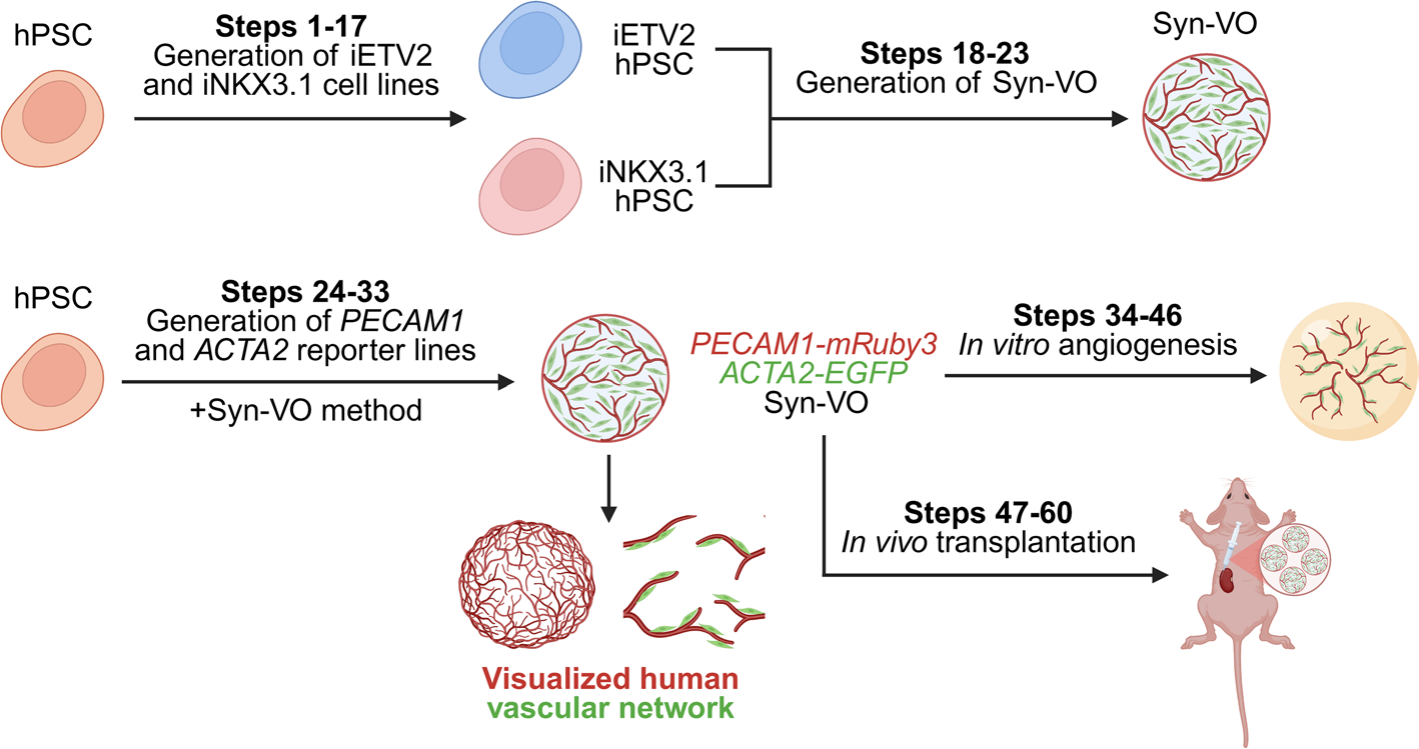
Schematic diagram of the timeline for generation and application of synthetic vascular organoids from hPSCs. This protocol is based on simultaneous orthogonal programming of hPSCs to generate endothelial cells and mural cells using 3D culture. The first step is the establishment of reliable iETV2 and iNKX3.1 hPSC lines. Synthetic vascular organoids (Syn-VOs) can be generated within 6 d. To visualize human vascular networks, *PECAM1-mRuby3* and *ACTA2-EGFP* hPSC reporter lines can be generated to label endothelial cells and mural cells, respectively. For formation of human vascular networks, Syn-VOs can be embedded in hydrogel or transplanted under the kidney capsule

**Fig. 2:**
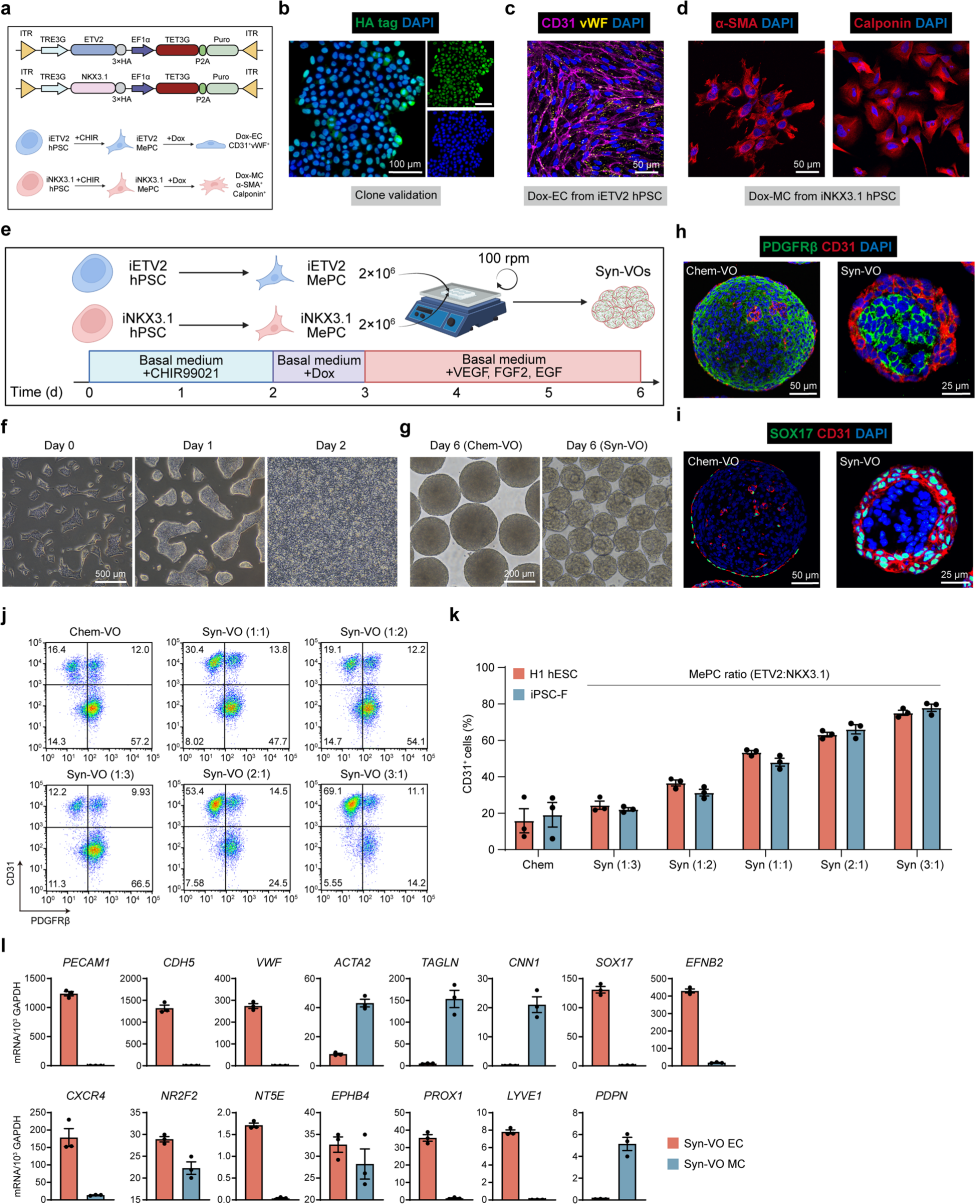
Generation of inducible hPSC lines and synthetic vascular organoids. **a**, Schematic illustration of iETV2 and iNKX3.1 constructs and the differentiation strategy toward Dox-ECs and Dox-MCs. **b**, Representative immunostaining images showing HA tag expression and nuclear localization after administration of Dox. **c**, Representative immunostaining images of Dox-ECs showing expression of CD31 and vWF. **d**, Representative immunostaining images of Dox-MCs showing expression of α-SMA and Calponin. **e**, Schematic illustration of the generation of synthetic vascular organoids (Syn-VOs). **f**, Representative bright-field images showing typical morphology at d0-2. **g**, Representative bright-field images showing the typical morphology of Chem-VOs and Syn-VOs. **h**, Representative immunostaining images of Chem-VOs and Syn-VOs showing the localization of CD31^+^ endothelial cells and PDGFRβ^+^ mural cells. **i**, Representative immunostaining images showing co-expression of CD31 and SOX17 in endothelial cells. **j**, Representative flow cytometry plots showing tuning of the endothelial cell to mural cell ratio. **k**, Quantification of the CD31.^+^ endothelial cell proportion across two hPSC lines (*n* = 3). **l**, RT-qPCR analysis of cell-type-specific markers in sorted ECs and MCs from Syn-VOs (*n* = 3). Scale bars: 25 μm (**h** (right panel), **i** (right panel)); 50 μm (**c**, **d**, **h** (left panel), **i** (left panel)); 100 μm (**b**); 200 μm (**g**); 500 μm (**f**)

This protocol enables the generation of VOs using a synthetic strategy. The first step is the successful establishment of iETV2 and iNKX3.1 hPSC lines (Figs. 1 and 2a). Upon Dox administration, immunostaining should reveal strong nuclear localization of the HA tag (Fig. 2b). Clonal purity can be evaluated based on the proportion of HA tag-positive cells, and clones with > 99% purity are optimal for subsequent differentiation. Prior to Syn-VO generation, the established lines should be independently induced toward Dox-ECs and Dox-MCs by adding Dox following MePC specification. Dox-ECs should express CD31 and vWF, whereas Dox-MCs should be positive for α-SMA and Calponin (Fig. 2c, d). For Syn-VO generation, hPSCs are first induced toward MePCs via Wnt activation using CHIR99021 for 2 days. MePCs are then dissociated, mixed, and cultured in suspension on an orbital shaker at 100 rpm. From d2-3, 0.5 μg/mL Dox is included to activate ETV2 and NKX3.1 expression. From d3-6, VEGF-A, FGF2, and EGF are added to promote EC and MC maturation (Fig. 2e). Chem-VOs can be generated in parallel as controls. A pronounced expansion of cells is typically observed at d1-2 (Fig. 2f). By d6, Syn-VOs generally display a smaller diameter than Chem-VOs (Fig. 2g). Syn-VOs can be characterized by immunostaining or flow cytometry. CD31^+^ ECs are predominantly localized to the outer layer, whereas PDGFRβ^+^ MCs reside in the inner regions (Fig. 2h). CD31^+^ ECs also co-express SOX17, indicating the arterial identity (Fig. 2i). The EC-to-MC ratio can be precisely tuned by mixing different proportions of iETV2 MePCs or iNKX3.1 MePCs, and the final cellular composition closely reflects the initial input ratio (Fig. 2j, k). In contrast, Chem-VOs typically contain only 10–30% CD31^+^ ECs, with greater variability across different cell lines and batches. Further RT-qPCR analysis confirmed the respective identities of ECs and MCs in Syn-VOs (Fig. 2l). Notably, Syn-VO ECs exhibited enriched expression of arterial markers (*SOX17*, *EFNB2*, and *CXCR4*), while displaying minimal expression of venous markers (*NR2F2*, *NT5E*, *EPHB4*) and lymphatic markers (*PROX1*, *LYVE1*, *PDPN*), confirming the specification toward an arterial lineage.

**Fig. 3:**
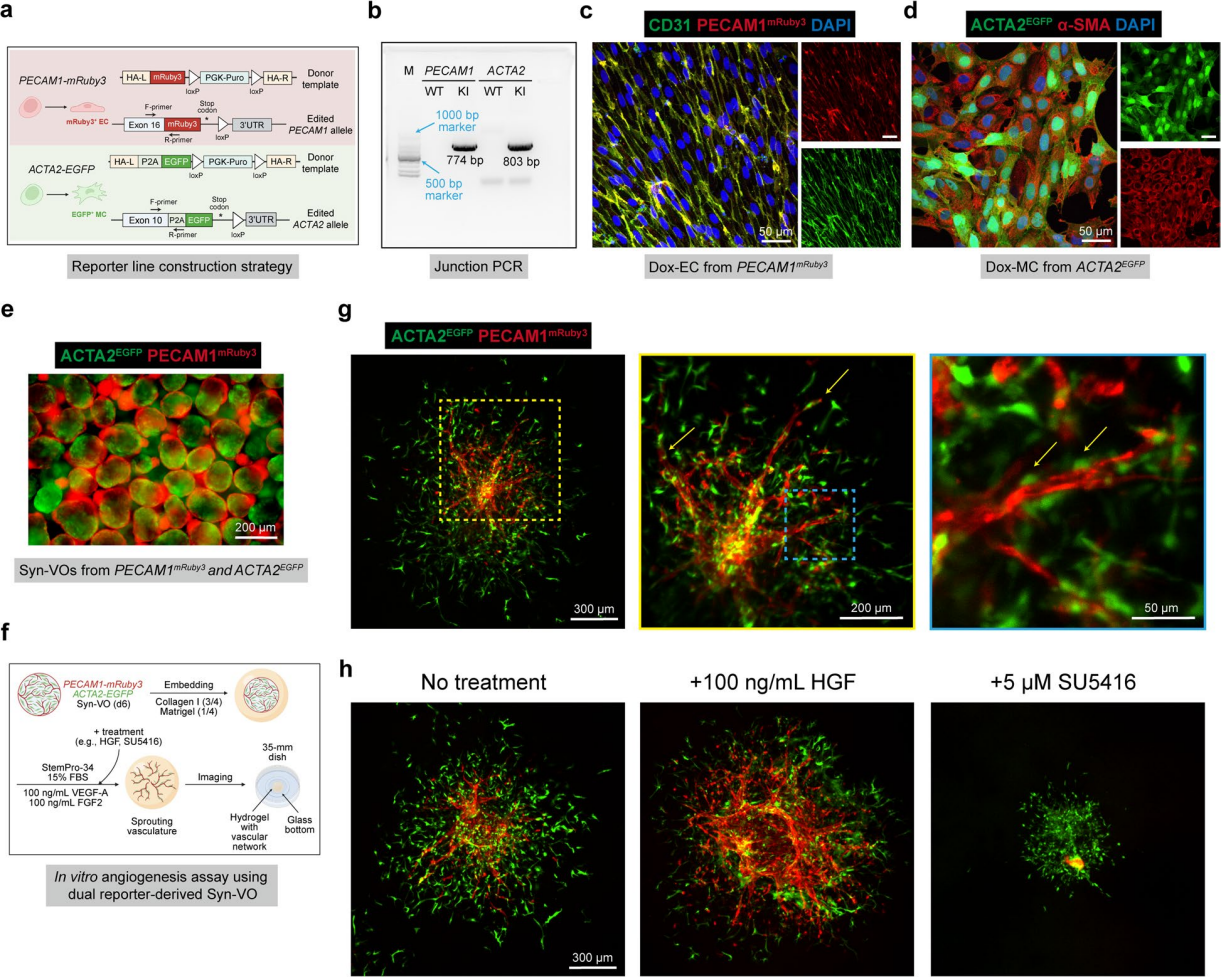
Construction of vascular reporter cell lines and in vitro angiogenesis assay. **a**, Schematic illustration of reporter line construction strategy. **b**, Representative junction PCR result of knockin clones. **c**, Representative immunostaining images showing co-localization of CD31 and mRuby3 signaling in Dox-EC. **d**, Representative immunostaining images showing co-expression of α-SMA and EGFP in Dox-MC. **e**, Representative fluorescent image of synthetic vascular organoids (Syn-VOs) derived from reporter lines. **f**, Schematic illustration of the in vitro angiogenesis assay. **g**, Representative fluorescent images of human vascular networks embedded in hydrogel, showing mRuby3^+^ vessels covered by EGFP.^+^ mural cells. **h**, Representative fluorescent images of human vascular networks treated with HGF or the VEGF inhibitor SU5416. Scale bars: 50 μm (**c**, **d**, **g** (right panel)); 200 μm (**e**, **g** (middle panel)); 300 μm (**g** (left panel), **h**)

Syn-VOs can be embedded in hydrogel or transplanted under the kidney capsule to induce the formation of human vascular networks. To facilitate efficient visualization, *PECAM1-mRuby3* and *ACTA2-EGFP* reporter hPSC lines can be generated to label ECs and MCs, respectively (Fig. 3a). Donor and sgRNA plasmids are available and have been validated across multiple hPSC lines. Correctly targeted clones can be identified by junction PCR, yielding a 774-bp band for *PECAM1* knockin and an 803-bp band for *ACTA2* knockin alleles (Fig. 3b). Reporter fidelity can be confirmed by differentiation into Dox-ECs and Dox-MCs, in which reporter fluorescence should co-localize with CD31 or α-SMA immunostaining (Fig. 3c, d). These reporter lines also enable real-time monitoring of Syn-VO formation (Fig. 3e). To induce vascular network formation in vitro, Syn-VOs at d6 are embedded in hydrogel and stimulated with high concentrations of VEGF-A and FGF2. This system supports drug screening for pro- or anti-angiogenic compounds (Fig. 3f). After 5 days, branched mRuby3^+^ vascular structures covered by EGFP^+^ pericytes should be observed (Fig. 3g). Addition of HGF further enhances branching, whereas inhibition of VEGF signaling with SU5416 abolishes network formation (Fig. 3h).

**Fig. 4:**
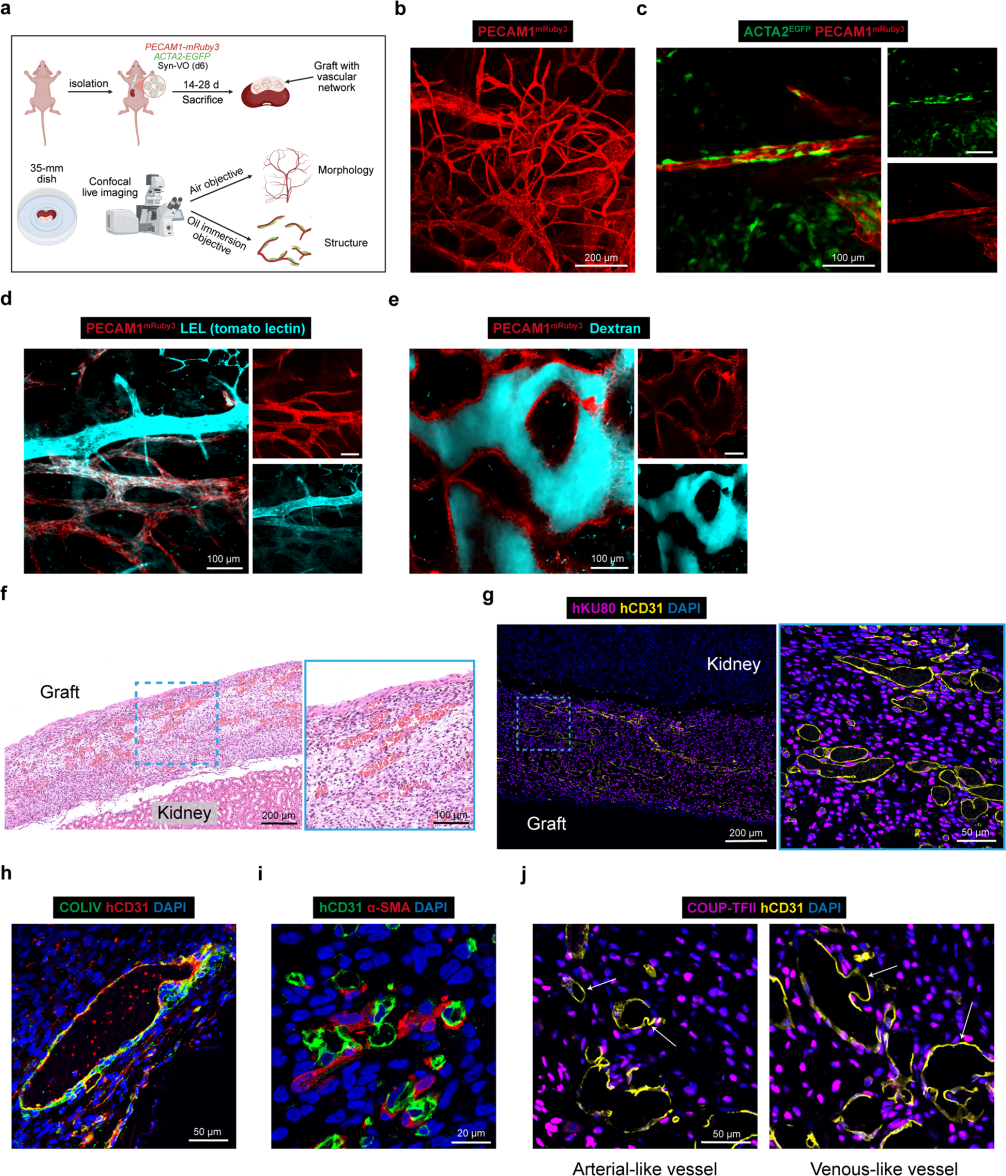
Transplantation of synthetic vascular organoids under kidney capsule. **a**, Schematic illustration of the transplantation and imaging strategy. **b**, Representative fluorescent image of the mRuby3^+^ human vascular network under kidney capsule. **c**, Representative fluorescent images of the mRuby3^+^ human vessel covered by EGFP.^+^ mural cells. **d**, Representative fluorescent image of human vessels perfused with tomato lectin. **e**, Representative fluorescent image of human vessels perfused with dextran. **f**, Representative H&E staining images on the graft and kidney, showing red blood cells within human vessels. **g**, Representative immunostaining images on human KU80 and human CD31 revealing engraftment of human vessels. **h**, Representative immunostaining images of type IV collagen and human CD31 showing basement membrane-enveloped human vessels. **i**, Representative immunostaining image on human CD31 and α-SMA showing mural cell coverage of human vessels. **j**, Representative immunostaining image on human CD31 and COUP-TFII showing the arterial and venous identity after transplantation. Scale bars: 20 μm (**i**), 50 μm (**g** (right panel), **h**,** j**); 100 μm (**c**, **d**, **e**, **f** (right panel)); 200 μm (**b**, **f** (left panel), **g** (left panel))

The injectable nature of Syn-VOs facilitates transplantation. Grafts can be analyzed by confocal microscopy 14–28 days post-transplantation (Fig. 4a). Highly branched mRuby3^+^ vascular networks form under the kidney capsule (Fig. 4b), and they are enveloped by EGFP^+^ pericytes (Fig. 4c). Intravenous injection of tomato lectin or dextran before sacrifice reveals perfusion of human vessels, demonstrating functional connection with host vasculature (Fig. 4d, e). Histological analysis using H&E staining shows vessels containing red blood cells (Fig. 4f). Immunostaining for human KU80 and CD31 confirms the distribution of human vasculature (Fig. 4g). The human vessels are surrounded by type IV collagen-positive basement membrane and covered by α-SMA^+^ pericytes (Fig. 4h, i). Following transplantation, a subset of ECs transitioned toward venous identity, a phenotypic shift potentially driven by the hypoxic microenvironment under kidney capsule or functional anastomosis with the host circulation. Consequently, both COUP-TFII^−^ arterial-like and COUP-TFII^+^ venous-like vessels were observed, underscoring the plasticity of ECs within Syn-VOs (Fig. 4j).

## Discussion

In summary, this protocol establishes a robust, highly reproducible, and versatile platform for generating Syn-VOs from hPSCs. The key features and technical advantages of this methodology lie in its application of orthogonal forward programming driven by the inducible expression of ETV2 and NKX3.1. Compared to conventional growth factor-based methods, which often suffer from line-to-line variability in differentiation efficiency, this approach exhibits remarkable consistency across hPSC lines, yielding organoids with well-defined cellular components. Consequently, this streamlined platform is ideally suited for high-throughput screening of pro- or anti-angiogenic drugs, as well as for modeling inherited vasculopathies using patient-derived lines.

Despite its robustness and flexibility, this protocol has several limitations. First, the generation of Syn-VOs relies on the inducible expression of transcription factors, which requires the prior establishment and validation of genetically engineered hPSC lines. This step increases the initial time investment and may not be readily accessible to laboratories without experience in genome editing or electroporation-based hPSC manipulation. Although reporter and inducible lines can be expanded and cryopreserved for long-term use, clonal variation may still influence differentiation outcomes and should be monitored by routine quality control.

Second, although Syn-VOs include ECs and MCs as the majority of vessel components, this system does not fully recapitulate the cellular diversity of native vasculature, including lymphatic ECs and immune cells. As a result, immune-vascular interactions or inflammatory responses cannot be modeled using this protocol alone and may require additional co-culture or in vivo approaches. In addition, the current Syn-VO system does not incorporate interactions with other somatic cell types. Given that vascular beds are highly specialized and exhibit marked phenotypic heterogeneity across different organs, future efforts should focus on developing organ-specific VOs. Such organotypic vascular models would enable more precise investigation of tissue-specific vascular disorders.

Third, the differentiation and assembly of Syn-VOs are optimized for a defined temporal window, and continued suspension culture beyond the recommended time frame results in a gradual loss of EC content. This phenomenon may be attributed to two potential mechanisms: first, MCs have higher proliferative capacity compared to ECs, leading to progressive overgrowth of MCs. Second, in the absence of TGF-β inhibitor in the S2 medium, ECs may undergo endothelial-to-mesenchymal transition (EndMT), shifting their lineage identity. Therefore, prolonged in vitro maintenance of Syn-VOs without ECM embedding is not supported by the current protocol. Further optimization of maintenance conditions, such as supplementation with serum components or additional factors such as TGF-β inhibitor SB431542, may be required to support long-term stability of Syn-VOs.

Lastly, the current Syn-VO protocol is optimized using the Dox1 (24 h Dox induction) scheme, which characteristically generates a highly homogeneous population of arterial-like ECs (CD31^+^SOX17^+^). While this homogeneity enhances batch-to-batch reproducibility, it presents a limitation for studies requiring distinct venous networks. However, as established in previous work, this platform possesses modular flexibility. By adopting alternative programming dynamics (such as the Dox3 scheme), Syn-VOs can have mixed or venous-like EC population to model venous EC-related biological processes (Gong et al. 2025).

## Data Availability

All data generated during this study are included in this article. The plasmids are available on Addgene or Wekwik. Requests for materials should be addressed to the corresponding author.

## Abbreviations

hPSC: Human pluripotent stem cell
ECs: Endothelial cells
EB: Embryoid body
ECM: Extracellular matrix
VOs: Vascular organoids
ETV2: Ets variant transcription factor 2
NKX3.1: NK3 homeobox 1
MCs: Mural cells
MePCs: Mesoderm progenitor cells
VEGF: Vascular endothelial growth factor
FGF: Fibroblast growth factor
TGF-β: Transforming growth factor beta
PDGF: Platelet-derived growth factor
2D: Two-dimensional
Dox: Doxycycline
mRNA: Messenger RNA
hESCs: Human embryonic stem cells
hiPSCs: Human induced pluripotent stem cells
3D: Three-dimensional
Syn-VO: Synthetic vascular organoid
Chem-VO: Chemical vascular organoid
sgRNA: Single guide RNA
PIK3CA: Phosphatidylinositol-4,5-bisphosphate 3-kinase catalytic subunit alpha
TEK: TEK receptor tyrosine kinase
MSCs: Mesenchymal stromal cells
BMP4: Bone morphogenetic protein 4
VEGF-A: Vascular endothelial growth factor A
DLL4: Delta like canonical Notch ligand 4
NOTCH3: Notch receptor 3
CDS: Coding sequence
DNA: Deoxyribonucleic acid
HA: Hemagglutinin
DAPI: 4',6-diamidino-2-phenylindole
EDTA: Ethylenediaminetetraacetic acid
DMSO: Dimethyl sulfoxide
EGF: Epidermal growth factor
DMEM/F12: Dulbecco's Modified Eagle Medium/Nutrient Mixture F-12
FBS: Fetal bovine serum
MW: Molecular weight
RT: Room temperature
PBS: Phosphate-buffered saline
BSA: Bovine serum albumin
PFA: Paraformaldehyde
CD31: Cluster of differentiation 31
PDGFRβ: Platelet-derived growth factor receptor beta
FACS: Fluorescence-activated cell sorting
EGFP: Enhanced green fluorescent protein
PCR: Polymerase chain reaction
α-SMA: Alpha-smooth muscle actin
HGF: Hepatocyte growth factor
KU80: Ku core protein p80
SOX17: SRY-box transcription factor 17
vWF: Von Willebrand factor
COL IV: Collagen type IV
FGF2: Fibroblast growth factor 2
CADASIL: Cerebral autosomal dominant arteriopathy with subcortical infarcts and leukoencephalopathy
CCM: Cerebral cavernous malformations
HHT: Hereditary hemorrhagic telangiectasia
EndMT: Endothelial-to-mesenchymal transition
